# Dental Anomalies, and Their Influence in the Production of Diseases of the Maxillary Bones

**Published:** 1860-01

**Authors:** A. M. Forget

**Affiliations:** Docteur en Médecine, Chevalier de la Légion d'honneur, Lauréat de l'Institute de France, Membre titulaire de la Société Médicale d'emulation de Paris; and Member of several other learned Societies, National and Foreign.


### AMERICAN JOURNAL

OF

### DENTAL SCIENCE.

Vol. X. NEW SERIES—JANUARY, 1860. No. 1.

### ORIGINAL COMMUNICATIONS.

### ARTICLE I.

*Dental Anomalies, and their Influence in the Production of Diseases of the Maxillary Bones.*—By A. M. FORGET, Docteur en Médecine, Chevalier de la Légion d'honneur, Lauréat de l'Institute de France, Membre titulaire de la Société Médicale d'emulation de Paris; and Member of several other learned Societies, National and Foreign. Prize Essay of the "Académie des Sciences, March 14, 1859." Translated for the Am. Jour. of Dental Science.

#### *Prefatory.*

THE bones of the face are subject to certain lesions of vitality and structure which are common to them with those of the other different parts of the body. These lesions are included in the general study of the diseases of the osseous system, and are subordinate to the same histological influences. Some, distinctly marked by the anatomical peculiarities which they present, and above all, by their origin, especially affect the maxillary bones; these we have exclusively to do with in this memoir.

To explain the anatomical and physiological reasons which predispose the jaws to the so frequent development of organic lesions which require the intervention of art, to demonstrate, by pathological anatomy, the causes which produce these lesions, the anomalies of nutrition, and the position of the teeth ; to show forth the primordial characters which enable the surgeon to trace these anomalies, and which permit him to arrive at the pathological consequences; and finally, if they already exist and reside in a more or less voluminous tumor of the jaw, to open a way to the diagnostic, which, in rendering the origin and nature of this tumor accessible and palpable, allow of a restriction of the operation simply to the diseased parts, and thus the preservation of the continuity of the bone, which the partial or total ablation would not sufficiently accomplish—such is the end which I have proposed to myself, and which from the public reward bestowed by the Academy of Sciences, I am led to hope I have attained.

### *Introductory.*

Of the different parts of which the living skeleton is composed, there is none which may more frequently be the seat of numerous and varied pathological productions than the maxillary bones, and particularly the inferior maxillary bone.

This peculiarity, if we consider that the morbid aptitudes of any organ of the economy whatever, are in respect to the complexity of its structure and the degree of vitality which it enjoys, would never surprise an attentive observer; it is the natural consequence of a double anatomical disposition proper to the maxillary bones, and to which an entire order of physiological phenomena is attached, for the accomplishment of which an organic activity and nutritive movement is created and sustained in their diaphysis, which is not partaken of in an equal degree by any other point of the osseous system.

Thus these bones offer a protecting canal to a vascular and nervous apparatus, receiving from them in exchange a very great number of nutritive vessels. The presence of the dental bulbs, moreover, which are arranged in the interior of the jaws, there to await their successive evolutions, and remain there till they are accomplished, act as a permanent cause of irritation and of congestion, the intensity and duration of which are in proportion to the slowness the difficulties and the anomalies of development of those osteides.

Now there are also morbid influences, remote, but not the less certain, to which we must refer these diseases, by which, like all organized products, the dental organs themselves are attacked at the different phases of their growth ; and after this isentirely achieved, slight and circumscribed, these diseases most generally pass unperceived, but in some cases, by their persistence, their generalization, and above all, by their extension to the alveolar tissue, they become the point of departure for deep seated osseous lesions for which the intervention of art is necessary.

The analysis of a great number of documents, at present in the possession of science, on the diseases of the jaws, has long since demonstrated to me the justice of the preceding etiological view, and I do not doubt but that whoever desires to study this question of pathogenesis from the same point of view, will be led to partake of my opinion, which will be justified, I hope, by this work which comprises two chapters—the first dedicated to the anomalies of nutrition, the second to the anomalous position of the teeth, and both having in view to demonstrate the connection between these anomalies and different morbid states of the maxillary bones.

### CHAPTER FIRST,

### *Anomalies of Nutrition and Development.*

Of all the observations which will figure in this chapter none seems to me to be more worthy of the first place than that which relates to a young patient whose case I have already had occasion to bring before the Society of Surgery; in a histological point of view, nothing can be more conclusive. It presents, in fact, the anatomical anomaly and the pathological lesion brought together and united in one case—a morbid duality most rare and unique in the human species, if I may judge from the impossibility of finding, in the various treatises of surgery, as well as in the collections of pathological anatomy, a single case which can be comparéd to it.

*Observation 1st.—Osteo-dental tumor, of the size of a large egg, encysted in the thickness of the inferior maxillary bone. Ulcerous inflammation of the parietes of the cyst. Numerous ossi-fluent fistulas. Resection of the left side of the body of the jaw, and of a portion of its branch. Guerison.*

During the first of the month of May, 1855, M. L., a dealer of the Point-a-Pitre, (Gaudaloupe,) presented his son to me, at the advice of Dr. Lerminier, a learned and experienced practitioner of that city, for the purpose of having him submit to an operation necessitated by disease of the inferior maxillary bone, the first indications of which had been observed at the age of five years.

*History of the Disease.*—At this period, the young L. experienced pains, accompanied by long intermissions, in the left side of the jaw, which continuing and becoming so aggravated, at the age of seven years he was obliged to submit to the extraction of two small molars, although they were perfectly sound, under the idea that they impeded the evolution of the second teeth. This operation produced marked relief; the intra-maxillary pains ceased, but soon after, a small, rounded, hard tumor of the size of a hazel-nut discovered itself upon the external face of the jaw, over the place from whence the teeth had been taken away.

Circumscribed, painless and stationary, the tumor did not make any sensible progress till the eighth year. The whole of the left side of the jaw then became tumefied, so much so that the planes of the bones were, according to the patient, hardened and rounded.

The absence of the large molars on the left side, although they were regularly developed on the right, was a striking attendant circumstance.

The morbid ampliation of the jaw was accompanied at intervals by a fluxionary movement of the gums, cheek, and the whole of the left side of the face. This fluxion, each time that it was produced, was very painful and resulted in sustaining and augmenting the tumefaction of the soft parts, so that the difference in volume of the two sides of the face became very apparent, and constituted a real deformity.

During the month of November, 1854, the swelling became quite large, active inflammation invaded the face of the jaw and the cervico-maxillary region. Antiphlogistic treatment was indicated, and two applications of leeches were made. Fifteen days after the inflammatory symptoms amended, purulent matter formed in the thickness of the cheek, for which an opening was spontaneously made, giving issue to a considerable quantity of fetid pus. The opening of this abscess remained fistulous; the ambient tissues being thin and ununited, and the bone denuded under them to a considerable extent.

*Actual State.*—Young L. is twenty years of age, strong and well developed, and is possessed of an excellent constitution; his general health has not suffered from the local affection with which he is attacked. This latter shows itself exteriorly by a large tumefaction of more than three times the natural size of the left cheek; it has assumed an ovoid form, and impressed a corresponding marked and eccentric development on the maxillary bone. In causing the patient to open his mouth—and this may be done with ease—we may perceive the whole of the left side of the bone, resembling a large turkey egg in form and volume. Thus it follows that the line of demarcation between the internal and external faces of the bone is obliterated, and is now described by a very apparent curve.

The tumor thus constituted is uniform, equal, and without creases or any irregular swelling upon its surface. Hard and resistant, it does not yield at every point to pressure, nor does it give the crepitating noise characteristic of the thinning of the osseous tissue. The exterior projection which it makes, hides the superior and lateral part of the neck, over which it extends several centimetres.

From the side of the mouth, the ampliation of the bone has forced the tongue from its proper direction, and the floor of the buccal cavity falls from left to right.

The alveolar border is singularly enlarged and does not show from the first bicuspid, which is regularly placed in its alveolus, any of the molar teeth where they should be seen. The tissue of the gums, of a deep red color, is thick and indurated. This tissue is interrupted and leaves an entirely naked, grayish, unequal, rugose surface, about as large as a twenty centime piece, which yields a sound like that of the crown of a tooth buried in the alveolus when struck by a metallic probe.

As to the limits of the disease, in front it does not pass the symphysis of the chin, and behind, by the aid of the finger, at the isthmus of the throat, it may be perceived at the posterior border of the branch of the maxillary bone where it is arrested. The upper part of the bone, as well as the articulating condyle, is healthy.

I would add, by way of completing this symptomatic explanation, that at the base of the tumor, several fistulous passages from the bone are to be found. The seat of an old purulent discharge, about two fingers in breadth, may be seen at the left labial commissure; this has caused the thin and violet-colored skin to come off for an extent of some four centimetres; finally, several of the sub-maxillary lymphatic ganglions are notably hypertrophied and indurated.

As to the functional troubles caused by the pathological condition which preceded, from having been slight at first they daily became more decided; thus phonation is embarrassed, mastication is painful and incomplete, deglutition is often only accomplished with difficulty, and respiration becomes difficult every time an inflammatory exacerbation attacks the tumor and the soft parts which confine it. In conclusion, the patient suffers from two very grave inconveniences, resulting from the very marked deformity of his face, and from the incessant flowing of a fetid pus proceeding from the complicated fistulas of ostéo-dental caries.

*Diagnosis of the Disease.*—After this exposé of the historical details of the disease, and the symptomatic traits which characterise it, the question which naturally presents itself to the mind is relative to the diagnosis, the solution of which prescribes the therapeutic indication to follow.

Now, an analysis of the preceding observation places one important fact strongly in relief, which, in my mind, touches the origin of the evil, and allows one, in consequence, if not to determine its more intimate character, at least to apprehend the initial point of departure. I refer to the relation existing between the appearance of the inter-maxillary pains and the time of life when the work of second dentition being in progress, the jaw became the centre of fluxion, determined and carried on by the excessive nutritive movement of which it was then the seat. Thus at five years of age young L. experienced the first painful attacks of his malady, of which nothing then indicated its future development and gravity. A little later, that is at seven years of age, precisely at the time when the growth of the dental bulbs has been completed by their alveolar eruption, the pains recur with such intensity that the extraction of two healthy teeth is judged necessary; the effect of this operation was only palliative, and it was soon followed by a new morbid manifestation in the appearance of a small tumor.

Let us add, if these remarks have not already established the histological connection I have in view, that this latter will soon become more evident if we look several years later when the absence of the larger molar teeth can be proven, their development never having taken place. Between this anatomico-physiological observation, the morbid accidents which have preceded it, and the evolution of the pathological fact which is now manifested by a voluminous tumor, it seems to me that something more exists than a mere coincidence; to me an intimate connection appears to occur, if not of direct causality, certainly of reciprocal influence.

But if it is easy to rise to the origin of the evil, is it equally so to determine upon its nature? This aspect of the problem unquestionably offers the greatest difficulties, although the proofs furnished by observation have led me to believe it not insoluble.

Thus, taking into consideration the slowly progressive mode of development of the tumor, the regular uniformity of its exterior aspect, the exact circumscription of its very clearly defined limits, the consistency and firmness of the different points of its surface, which is bounded on all sides by an osseous frame work, I do not hesitate to state the existence of a pathological product which has taken birth in centre of the body of the maxillary bone, the eccentric development of which had followed the growth of this product in such a manner as to enclose all parts of it as in a true cyst; this arrangement in all respects resembles that which I have frequently observed and which I described during 1840 in my inaugural thesis,* under the designa-

* Recherches sur les kystes des os maxillaires et leur traitement, 1840.

tion of *osseous cyst* enclosing a solid product. As to the intimate composition of this cyst, I did not believe that it was possible to have a very precise knowledge of it; also, without pronouncing upon the nature of the anatomical elements which constituted the pathological aggregate, I confined myself to diagnosticating the existence of an oméomorphous tissue at the centre of the maxillary bone ; considering the hypertrophy of the cervical ganglions as the result of a sympathetic irritation and not as the symptom of the cancerous infection already in progress. The former (the sympathetic irritation) was hardly admissible in the presence of the flourishing health of M. L. If the disease with which he was attacked had been characterised by this malignity it would have resulted differently, and in the space of twelve years which have elapsed since its appearance, it would have produced those more considerable local disorders and would have exercised a deleterious influence upon the physiological development, and likewise upon the constitution of the subject, the effects of which would not have been so slow to manifest themselves.

The question of diagnosis to us is always too grave in such a case and its solution of too high a practical interest for me to consent to report simply my own judgment. I therefore called in two of my colleagues of the Society of Surgery, MM. Michon and Denonvilliers, who, after examining the young L., partook of my opinion upon the mode of development of his disorder, its nature presumed, if not evident, and upon the necessity of a resection of a part of the body of the jaw, an operation which I performed on the 16th of June, 1855, with their assistance and in the presence of MM. Pinel de Golleville, Mongeal and of M. Felix Baudoin, a distinguished student of the hospital of Paris.

*Operation.*—The patient was placed upon a bed on his right side, the head elevated and resting upon a tolerably hard pillow. I commenced the operation, situating myself in front of the patient, after having submitted him to the inhalation of chloroform. In order to turn the base of the tumor I commenced an incision in front of the base of the ear and extended it to a distance of about one centimetre from the projection of the chin on the right side of the jaw. By this I circumscribed a large flap, which, when I had dissected and reversed it upon the cheek-bone, was found to consist of the whole thickness of the cheek and a portion of the lower lip. The dissection of the soft parts was unavoidably long, as well on account of the close attachments of these to the surface of the bone, as on account of the ligatures necessitated by the section of several arterial branches.

The flap being fastened up, I sawed the bone in front upon the alveolus of the canine tooth which I had previously removed. Behind, the saw intersected the maxillary at the point comprised between the angle formed by the branch of the bone and the origin of the dental canal. For executing this part of the operation, I used a chain-saw; the play of this instrument was interrupted, however, in front by an unforeseen obstacle which nevertheless I eventually surmounted; this was the presence of a tooth placed horizontally in the thickness of the bone and precisely under the alveolus occupied by the track of the instrument.

*Excision of the Tumor.*—When the osseous tumor was isolated by these two applications of the saw, I proceeded to detach the inferior parts by dividing all that part of the floor of the mouth corresponding and adhering to the internal face of the jaw, with a bistoury. In order to accomplish this, an assistant having seized it with flat-bladed pincers, and holding it turned inside out, I made an incision, from front to back, through the muscles of the sub-maxillary region, taking care constantly to turn the cutting edge of the bistoury against the tumor, thus avoiding the base of the tongue, and, above all, the anterior pillar of the veil of the palate which, as I remarked in the symptomatic exposé of the observation, marked, behind and within, the limits of the morbid development of the jaw. This division of the lower tissues necessitated several ligatures around the divided branches of arteries, after the tumor was raised; I also introduced a small ball of wax into the orifice of the open dental canal in the cut face of the bone and thus put a stop to the hemorrhage arising from the dental artery.

In order to complete the operation and leave in the wound no tissue of a suspected character, I dissected and raised up two lymphatic ganglions situated under the tongue and above the genio-hyoid muscles and the anterior belly of the digastric muscle. This done, I waited some moments before proceeding to dress the wounds, which I commenced only when I was assured that all danger from hemorrhage was over.

The flap, left to itself, by its own weight, entirely recovered the solution of continuity. It was also easy to bring the edges of this together and maintain them united by means of the twisted suture, the points of which I arranged to the number of sixteen, in such a manner as to leave an opening in the lower part through which I placed a thread ligature and by which the purulent fluids found a permanent and easy issue. A perforated compress and a cerate plaster, a pledget of lint soaked in cold water, and over all a chin-piece completed the apparatus applied.

*Remark.*—To effect this resection by the process I have made use of, and which has for its object to preserve the lowerlip intact instead of dividing it in all its height by commencing an incision upon its free edge, it is important to take care in the formation of the flap; it is necessary to give sufficient extent to the incision which determines the length of the flap to allow of the introduction and use of the chain-saw without impediment. If it were found necessary to formularise a rule bearing upon this point, I should say that instead of arresting one's self on the limits of the tissue to be removed, it would perhaps be better to extend the incision in front as well as behind, two or three centimetres beyond. The dissection of the soft parts also might be carried farther and by this a much greater separation of the borders of the solution of continuity might be obtained at their angles ; this would permit the surgeon to maneuver easily with the chain-saw.

*After the Operation.*—During the operation, the pains of which were but feebly mitigated, the continued inhalation of chloroform having been rendered impossible from the nature of the case, the patient showed great courage. After it was over he was faint, and very pale ; his pulse slow and feeble. Having been taken to his bed, his head elevated and reposing on a horse-hair pillow, he was not long in going to sleep. In an hour he awoke and vomited a large quantity of blood without any effort to expectorate ; until evening he rested in a somnolent and prostrate state ; then, during two hours, several spoonfuls of bouillon were given to him and in the interval wine-water. At eight in the evening the reaction had fairly taken place ; the skin was warm, the face animated, and the pulse indicated 106 pulsations a minute.

*The day after the Operation* (17*th of June*).—After an agitated night, the fever became intense, (pulse at 130.) The patient had not urinated for twenty-four hours ; the epigastric region was tense, no pain, and percussion left no doubt as to the considerably enlarged state of the bladder. I made an effort to use the catheter but the spasm of the urethra rendered it so very painful that I did not insist ; I prescribed camphorated oil, however, upon the hypogastrium, to be followed by the application of a large cataplasm. This had the result which I intended ; at the end of two hours the patient urinated freely.

On removing the covering the wound appeared in a satisfactory condition ; no tension, swelling, or isolated redness appeared along the line of the sutures. Adherences having taken place at the point which I had intended to leave open for the free escape of fluids, I destroyed them with the extremity of a probe ; I also moved the pin nearest to the symphysis of the chin. The same dressing was applied which was used the day before ; for diet-drink, gum-water was directed with instructions to persevere in a strict diet.

18*th of June.*—The febrile movement continued intense through the night; the pulse at 135. The patient has been very restless and has not slept. Now the pulse indicates 120 pulsations, small, hard, and depressible: the heat of the skin is moderated ; the patient signifies that he feels better, but complains most of the fatigue caused by the constant spitting of bloody saliva. I removed all of the pins except three at the small part of the wound : a simple dressing is substituted for the application of compresses soaked in cold water. Chicken broth is directed, and an opiate drink for the night.

19*th of June.*—The fever has diminished ; pulse at 90 ; I removed the three last pins, and by moderate pressure directed from the base towards the perimeter of the flap, I caused an abundant flow of pus of good quality through that point of the wound where the ligatures were placed. Prescribed broth, a feculent potage.

20*th of June.*—Condition same as yesterday ; the night has passed calmly with prolonged sleep. The edges of the wound have completely united along its anterior third.

21*st.*—*Sixth day after the Operation.*—Sensibility exist in the neighborhood of the wound without any elevation of the pulse.

22*nd.*—During the afternoon of yesterday, the patient had a severe chill with violent headache and coldness of the lower extremities. Fever followed the chill and the pulse rose to 130 ; it is now slightly reduced. In removing the dressing I found the cheek tumefied, hot, red and painful to the touch, with all the symptoms of an erysipelas in process of development. As to the erysipelas, as the evident sign of gastric embarrasment to which I apprehended a certain degree of gastro-intestinal irritation was connected, I combated it exteriorly by the use of mercurial ointment followed by collodion, the first having been lost interiorly, by saline purgatives and diluent and acidulated drinks.

*24th and After.*—Notwithstanding the treatment employed, the erysipelas continued its invading march, causing at first very intense febrile excitement. Starting from the left cheek, it successively attacked the wing of the nose, the eyelids, the temple and that hairy portion of it which should be shaved, finally limiting itself behind along the line of the occipito-parietal suture. From the left side it passed to the right and appeared upon the other cheek having taken ten days in accomplishing its peregrinations. This circumstance of the case terminated, however, convalescence took place easily and rapidly. Reparative aliment, given with prudence, quickly restored the patient's vigor. For several days this consisted of soups, juices of meats, jellies, and a little later I permitted solid food. On the eighth day following the operation, young L., could easily eat bread, meat, and other articles which require efforts of mastication.

Towards the last of July, I presented M. L. to the Society of Surgery. The cicatrix of the operation was linear, the face showing no appreciable deformity. Looking at this young man in the face, one would be unable to perceive that he had lost so considerable a portion of his lower jaw.

(To be continued.)

